# Beyond Translation Initiation: Translational Elongation, Decoding Fidelity, and Ribosome Surveillance During Branched-Chain Amino Acid Deprivation, with a Focus on Mammalian Systems

**DOI:** 10.3390/biology15181629

**Published:** 2026-09-16

**Authors:** Mutsuki Ojima, Manami Matsuura, Miwako Deguchi, Dong-Ho Kim

**Affiliations:** 1 Department of Nutrition, Graduate School of Human Life and Ecology, Osaka Metropolitan University, 2-1-132 Morinomiya, Joto-ku, Osaka 536-8525, Osaka, Japanmdeguchi@fc.ritsumei.ac.jp (M.D.); 2 College of Sport and Health Science, Ritsumeikan University, 1-1-1 Noji-Higashi, Kusatsu 525-8577, Shiga, Japan

**Keywords:** branched-chain amino acids, aminoacyl-tRNA, ribosome stalling, ribosome collision, mistranslation, GCN2, integrated stress response, ribosome-associated quality control

## Abstract

In mammalian systems, translational responses to amino acid limitation are commonly framed as mechanistic target of rapamycin complex 1 (mTORC1) inhibition and activation of the general control nonderepressible 2 (GCN2)–eukaryotic initiation factor 2α (eIF2α) arm of the integrated stress response. Suppressing initiation, however, does not immediately relieve aminoacylated transfer RNA shortages for ribosomes already engaged on messenger RNA. Transfer RNA charging and ribosome profiling reveal codon- and isoacceptor-specific elongation constraints. Across mouse NIH3T3 and several human cell systems, leucine deprivation often appears initiation-dominant but can produce UUA-biased pausing and frameshifting in selected cancer cells. Valine deprivation can prolong decoding at all four valine codons, whereas separate isoleucine-deprivation studies report AUU/AUC-selective slowing, a cytoplasmic isoleucyl-tRNA synthetase 1 (IARS1)-linked isoleucine-to-valine signal in exogenous reporter peptides, and an isoleucine-to-methionine substitution signal of unresolved mechanism. Ribosome slowing can engage global, transcript-local, and quality-control feedback, but the inputs are stress- and system-dependent. We propose a two-layer kinetic framework separating (i) A-site competition among correct decoding, substitution, and abortive exit from (ii) collision formation set by ribosome influx and dwell time. It keeps protein quantity, sequence fidelity, retained function, and proteostasis cost separate, and defines a matched measurement roadmap from metabolite flux to protein function.

## Simple Summary

Mammalian cells make proteins from amino acids, and this process depends on an adequate supply of each amino acid. Amino acid limitation is often described mainly as causing cells to make less protein. This review explains why that is not the full picture. When amino acid supply falls, ribosomes—the molecular machines that assemble proteins—may slow down and, in some settings, collide with one another. In some circumstances, the translation machinery may even incorporate a different amino acid, producing a protein with an altered sequence. The branched-chain amino acids leucine, valine, and isoleucine share chemical and metabolic features, yet limitation of each can produce a distinct translational response. These differences may be relevant to nutrition, aging, and diseases such as cancer, in which cells often encounter an uneven amino acid supply. In short, a shortage of one amino acid can do more than reduce how much protein is made: it can change what is made and whether the resulting protein still functions correctly.

## 1. Introduction: Amino Acid Limitation Beyond Translation Initiation

In mammalian cells, protein synthesis is one of the largest consumers of nutrients and energy. When amino acids run short, cells must therefore cut biosynthetic demand quickly while still making the proteins needed for adaptation. Here, amino acid limitation means reduced amino acid supply relative to cellular demand; amino acid stress refers to the resulting challenge to homeostasis and the responses it elicits. These terms describe a nutritional condition and its cellular consequences, respectively, rather than two separate perturbations. The canonical framework emphasizes translation initiation: reduced amino acid availability can inhibit mechanistic target of rapamycin complex 1 (mTORC1) and activate the general control nonderepressible 2 (GCN2)–eukaryotic initiation factor 2α (eIF2α) arm of the integrated stress response (ISR). Leucine illustrates this logic particularly well: Sestrin2 directly binds leucine and helps relay leucine availability to mTORC1 [1,2]. GCN2 phosphorylates eIF2α, suppressing bulk initiation while favoring the synthesis of stress-responsive proteins such as activating transcription factor 4 (ATF4) [3]. Selective ATF4 synthesis during eIF2α phosphorylation is mediated by delayed reinitiation at upstream open reading frames in its messenger RNA (mRNA) [4].

Initiation control is necessary, but it is not sufficient to describe what a translating ribosome experiences. When nutrient supply falls, many ribosomes are already engaged on mRNAs, and their forward movement depends on delivery of the appropriate aminoacylated transfer RNA (aminoacyl-tRNA). Reduced charging can increase local dwell time [5,6]. Persistent delays can allow trailing ribosomes to collide [7,8], engage ribosome-associated quality control (RQC) [9,10], or alter decoding fidelity [11]. Codon-specific dwell-time changes can remain detectable even while global initiation declines [12], and collisions can trigger transcript-local initiation feedback [13,14]. Calibrated monosome, disome, and trisome measurements are required to distinguish these states [15,16,17,18].

This distinction changes the question from “is translation on or off?” to “how is ribosome flux redistributed when a specific amino acid becomes limiting?” The outputs are also multiple. How much full-length protein is made, how accurate its sequence is, how much of it stays functional, and how much strain it places on proteostasis need not change together. A cell may preserve apparent protein output while increasing amino acid substitutions, or it may suppress initiation sufficiently to prevent collisions while producing less protein.

Branched-chain amino acids (BCAAs)—leucine (Leu), valine (Val), and isoleucine (Ile)—offer a useful comparative system. They share structural features and portions of their metabolic handling, yet recent mammalian studies report qualitatively different effects on tRNA charging, codon dwell time, and fidelity [5,11,12]. The primary focus is mammalian translation, with direct BCAA-deprivation evidence drawn mainly from mouse and human cell systems and physiological context from rodent dietary studies and human nutrition. Selected bacterial, fungal, and nematode studies provide mechanistic context; their organisms are identified where discussed, and their findings are not assumed to establish mammalian BCAA responses. This review integrates those studies with mechanistic work on GCN2 activation, collided ribosomes, the ribotoxic stress response (RSR), and ribosome-associated quality control. The central claim is deliberately limited: BCAA deprivation reveals that initiation, elongation, fidelity, and surveillance are coupled layers of one resource-allocation problem. It does not imply that every amino acid limitation state produces collisions, mistranslation, or RQC. It should also be recognized at the outset that, as detailed in Section 3, the independent primary biological evidence base remains narrow and much of the field still relies on a small number of mammalian systems.

This narrative synthesis prioritizes direct BCAA-deprivation experiments for biological comparisons. Studies of other amino acids, stressors, and organisms are used to evaluate plausible mechanisms and measurement limitations. Reanalyses are distinguished from independent biological datasets. The framework in Section 7 integrates these observations into explicit assumptions, traffic predictions, and additional mechanistic hypotheses.

Table 1 distinguishes pausing, stalling, queueing, collision, quality-control engagement, functional deficiency, and adaptive translation by their experimental evidence. These ribosome states and responses should not be treated as synonyms. We use pausing for an inferred increase in relative ribosome dwell time or occupancy at a defined position. Stalling denotes a more persistent delay that impedes elongation flux. A collision occurs when a trailing ribosome reaches the delayed ribosome. Quality-control engagement requires downstream recognition, ubiquitination, splitting, rescue, decay, or nascent-chain processing. Monosome profiling can support an inference of slower decoding, but it does not by itself demonstrate collision or RQC [12,15,16,17,18].

## 2. From Amino Acid Supply to Aminoacyl-tRNA Availability

### 2.1. Metabolic Supply, Compensation, and Decoding Resources

The amino acid pool available for translation reflects both concentration and the balance of supply and use. Uptake, protein degradation, and inter-organ exchange replenish amino acid pools, whereas incorporation into protein and amino acid catabolism deplete them. Mammalian BCAAs depend on uptake and recycling; reversible transamination can regenerate a BCAA from its corresponding branched-chain keto acid but does not provide de novo synthesis of its carbon skeleton. Aminoacyl-tRNA synthetases (aaRSs) channel available amino acids into tRNA charging along the protein-synthesis pathway, rather than constituting a separate, additive demand [19,20,21,22]. A bulk intracellular amino acid measurement can therefore stay stable while the aminoacylated fraction of its cognate tRNAs changes. Conversely, a low extracellular concentration does not establish that the correctly aminoacylated tRNA is limiting at the ribosome.

Compensation can uncouple medium composition from ribosomal supply. Under Leu-free conditions, human *KRAS*-mutant cancer cells can use macropinocytosis followed by transcription factor EB (TFEB)-dependent lysosomal degradation of extracellular albumin to recover mTORC1 activity and growth. In nutrient-deprived mouse embryonic fibroblasts, lysosomal protein degradation maintained charging of most tRNAs even as tRNA^Gln^ became limiting [23,24]. Neither study examined BCAA codon decoding, but both show why removing an amino acid from the medium is not the same as limiting it at the ribosome.

tRNA charging converts this metabolic balance into a decoding resource. Each amino acid is served by one or more tRNA isoacceptors that differ in abundance, codon recognition, modification, and kinetic demand [25,26,27]. Selective isoacceptor charging was predicted theoretically [27], demonstrated during starvation in *Escherichia coli* [28], and linked to selective translation in human HEK293 cells [29]. Regulation also involves tRNA expression and modification, although key mechanistic evidence comes from nonmammalian models. In the yeast *Saccharomyces cerevisiae* and the nematode *Caenorhabditis elegans*, loss of wobble modifications disrupted codon-specific elongation and proteome integrity [30]. In *S. cerevisiae*, stress-responsive tRNA modifications biased translation toward selected transcripts during oxidative stress [31]. The relevance of tRNA modification pathways to mammalian proteostasis and human disease has been reviewed [32]. One amino acid therefore does not correspond to a single, uniform decoding pool.

Nutrient-sensing pathways exchange information rather than operating as isolated detectors. GCN2 can contribute to mTORC1 responses to glutamine and other amino acids. Conversely, a cell’s capacity for metabolic compensation can influence whether Sestrin2 is induced and mTORC1 is suppressed, and this capacity varies among cell types [33,34,35]. These findings do not establish direct activation of GCN2 by Sestrin2. mTORC1 output and GCN2–eIF2α signaling should therefore be measured independently in the same system, rather than inferring one pathway’s activity from the other.

Leucyl-tRNA synthetase 1 (LARS1) provides a particularly relevant bridge between aminoacylation and nutrient signaling. In a proposed leucine-sensing pathway, leucine-bound LARS1 interacts with RagD and regulates Rag GTPase cycling to promote mTORC1 activation, while Sestrin2 acts through the GATOR2–GATOR1 signaling module (GATOR, GTPase-activating protein activity toward Rags) [36,37,38]. These studies do not establish that one sensor universally replaces the other; instead, they illustrate why LARS1 aminoacylation activity, leucine sensing, and mTORC1 output should be measured in the same system.

These metabolic and signaling inputs converge on isoacceptor-specific decoding resources. Differences among isoacceptors explain why synonymous codons can respond differently to the deprivation of the same amino acid. Dwell time rises most when the tRNA that decodes a codon efficiently can no longer keep pace with ribosome demand. Near-cognate competitors, wobble decoding, and tRNA modifications can further alter the delay [6,25,26,39]. Codon occupancy should therefore be read alongside isoacceptor-resolved charging data whenever such data can be obtained.

### 2.2. Initiation and Elongation Are Coupled by Ribosome Load

**Figure 1:**
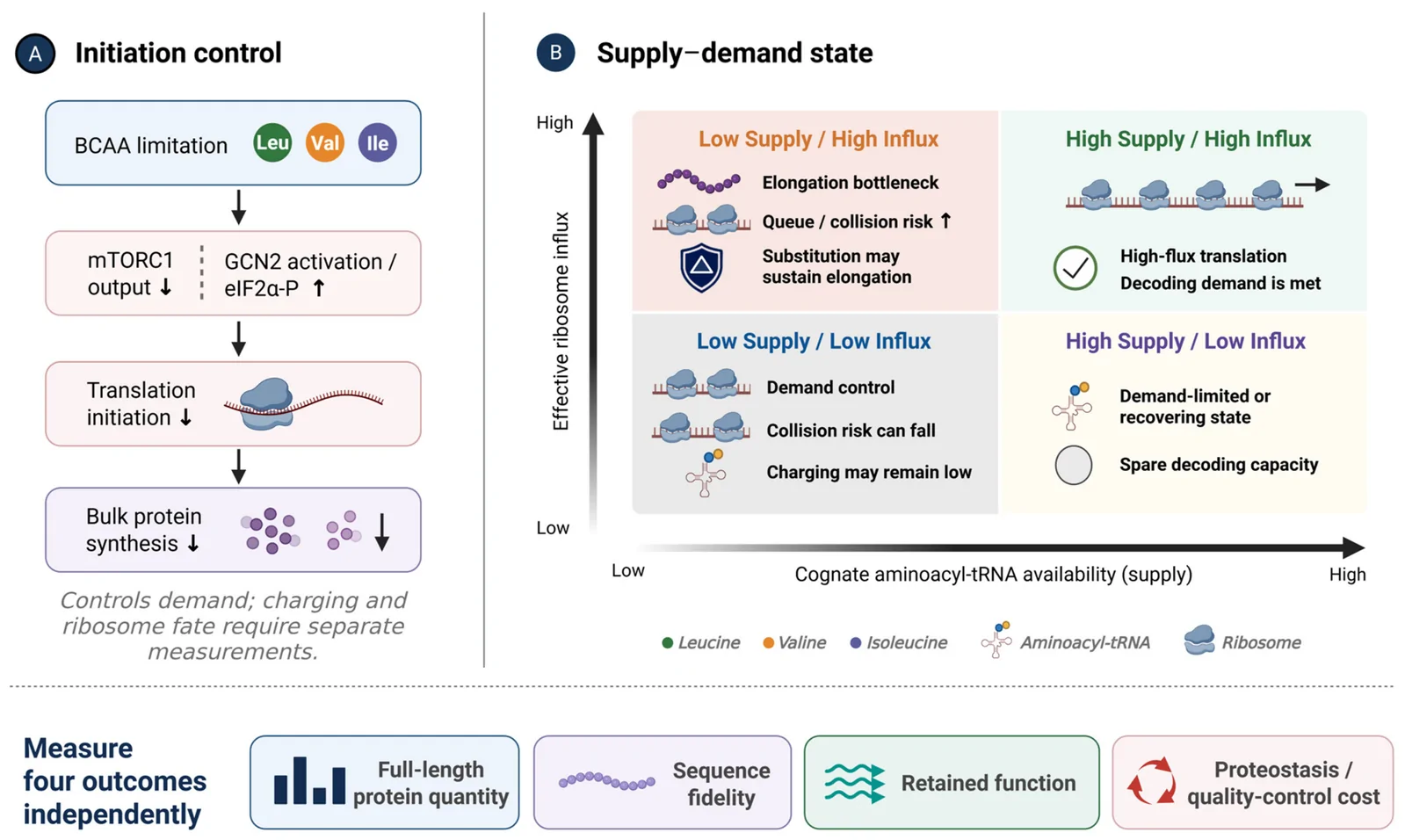
From an initiation-centered model to a multidimensional supply–demand framework. (**A**) Classical amino acid-stress models emphasize inhibition of mechanistic target of rapamycin complex 1 (mTORC1) signaling and activation of the general control nonderepressible 2 (GCN2)–eukaryotic initiation factor 2α (eIF2α) pathway, which reduce translation initiation and bulk protein synthesis. These responses control demand but do not by themselves define aminoacyl-tRNA charging, ribosome fate, or product quality. (**B**) Translational state depends on cognate aminoacyl-tRNA supply relative to effective ribosome influx. Low supply with low influx reflects demand control that can reduce collision risk without restoring charging, whereas low supply with high influx creates an elongation bottleneck that increases queueing and collision risk, while productive substitution may sustain elongation. High supply with low influx represents a demand-limited or recovering state with spare decoding capacity, whereas high supply with high influx supports high-flux translation. Functional deficiency can therefore arise when charging fails to meet current demand even though free amino acid remains detectable. Full-length protein quantity, sequence fidelity, retained function, and proteostasis or quality-control cost must be measured independently. The quadrants are conceptual states rather than fixed assignments for Leu, Val, Ile, or a particular cell type. Connecting arrows denote the indicated progression; ↑ and ↓ denote increases and decreases, respectively. Axis arrows indicate increasing supply or influx, and the arrow along mRNA indi-cates translation direction. Abbreviations: BCAA, branched-chain amino acid; mTORC1, mechanistic target of rapamycin complex 1; GCN2, general control nonderepressible 2; eIF2α, eukaryotic initiation factor 2α; eIF2α-P, phosphorylated eIF2α; Leu, leucine; Val, valine; Ile, isoleucine. Adapted from a figure created in BioRender. Kim, D. (2026). https://BioRender.com/ai2vcvx.

mTORC1 and GCN2 do more than control a common translation switch: they couple initiation to the supply–demand balance experienced by elongating ribosomes (Figure 1). GCN2–eIF2α signaling lowers ternary-complex availability and initiation, while mTORC1 regulates cap-dependent initiation and other anabolic outputs [3,34]. Under amino acid limitation, GCN2 activation and mTORC1 inhibition can both reduce the number of ribosomes entering coding sequences and thus demand for scarce aminoacyl-tRNAs. When ribosome load falls, ribosomes sit further apart and collide less often. When initiation stays high while a cognate aminoacyl-tRNA pool collapses, queues form instead. Initiation and elongation must therefore be inferred jointly [40,41].

Feedback operates at different spatial scales. GCN2–eIF2α mediates a global reduction in initiation, whereas endothelial differentiation-related factor 1 (EDF1) detects collided ribosomes and recruits the GRB10-interacting GYF protein 2 (GIGYF2)–eIF4E homologous protein (4EHP) complex to inhibit further initiation in cis on the affected mRNA [13,14]. This transcript-local brake can slow new ribosome influx without shutting translation down across the whole cell.

This coupling helps explain why severe amino acid depletion does not always produce the largest codon-specific pause signal. A strong initiation response can reduce ribosome influx enough to mask or relieve an elongation bottleneck. The apparent absence of a pause therefore does not demonstrate that cognate tRNA charging was preserved. It may indicate successful demand control. Darnell and colleagues made this point experimentally by contrasting Leu and Arg limitation, which produced different degrees of tRNA charging loss, pausing, and protein output [5] (Section 3.1).

At the organism level, this feedback is embedded in tissue exchange. Loss of GCN2 in mice during dietary Leu deprivation, for example, can preserve liver protein synthesis at the expense of skeletal muscle mass [42]. Local translation can therefore be sustained by redistributing systemic resources rather than by removing the underlying limitation. This distinction becomes essential when cell-culture starvation is used to interpret dietary interventions. Figure 1 summarizes the shift from an initiation switch to a supply–demand state space and separates four downstream outputs.

## 3. Distinct Translational Phenotypes of Leucine, Valine, and Isoleucine Deprivation

**Figure 2:**
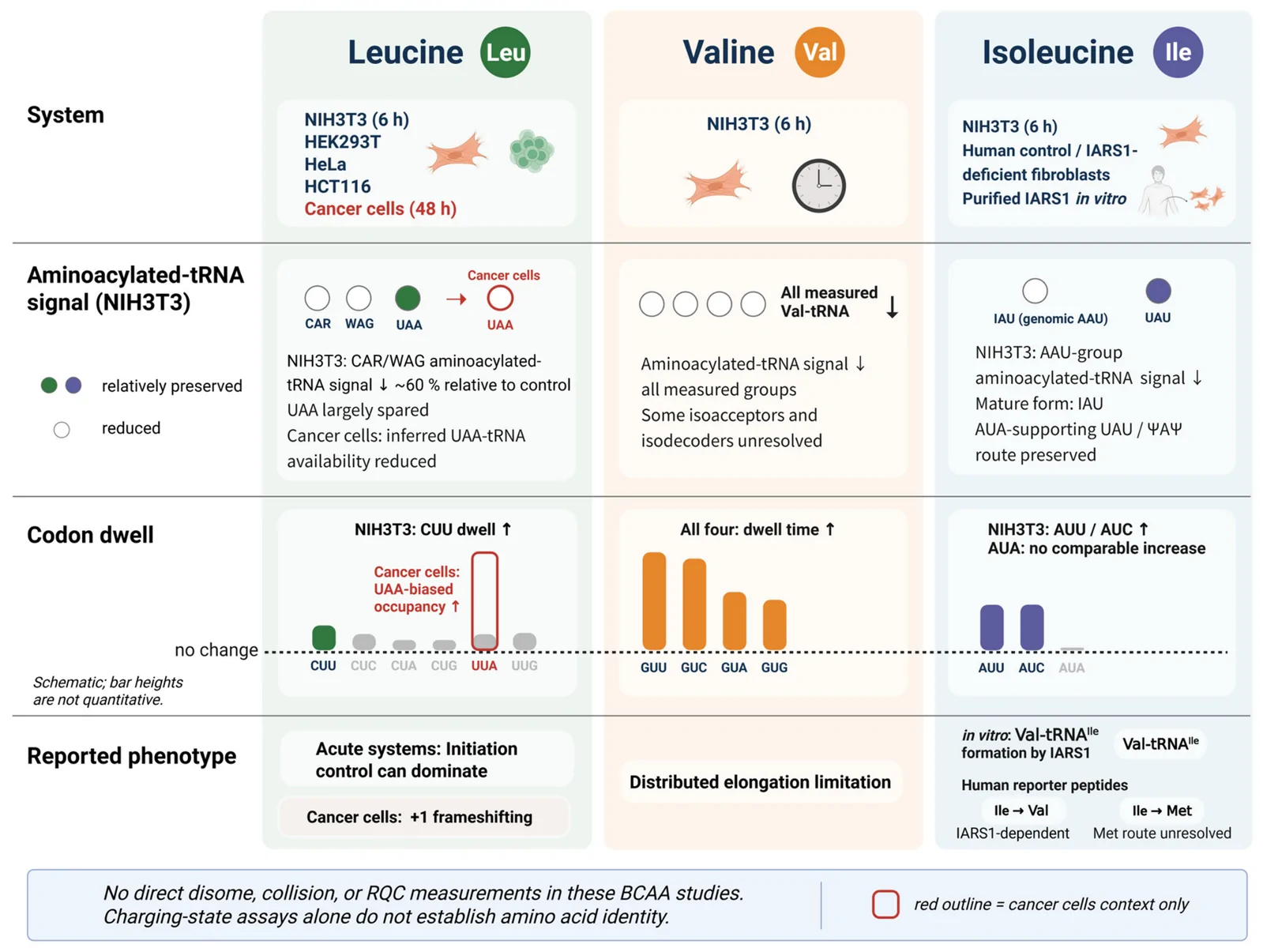
System-dependent translational phenotypes during branched-chain amino acid deprivation. The schematic integrates six-hour deprivation in mouse NIH3T3 fibroblasts [12], studies in human HEK293T, HeLa, and HCT116 cells [5], prolonged Leu deprivation in selected human cancer cells [43], and control and IARS1-deficient human fibroblasts [11]. During Leu deprivation, acute NIH3T3 cells showed an approximately 60% reduction in the aminoacylated tRNA^Leu^(CAR/WAG) signal relative to full-medium controls, relative preservation of tRNA^Leu^(UAA), and a significant dwell-time increase only at CUU [12]. By contrast, selected cancer-cell systems examined after 48 h showed UUA-biased occupancy and +1 frameshifting [43]. Val deprivation reduced charging across all measured Val-tRNA groups and increased dwell-time estimates at all four Val codons, consistent with a distributed elongation limit [12]. Ile deprivation reduced charging of the genomic tRNA^Ile^(AAU)/mature IAU group while relatively preserving the AUA-supporting UAU/ΨAΨ route and produced greater slowing at AUU/AUC than at AUA [12,46]. Separate evidence showed Val-tRNA^Ile^ formation by purified IARS1 in vitro and Ile-to-Val signals in cellular reporter peptides; an Ile-to-Met substitution signal was also detected, but its mechanism remains unresolved [11]. The matrix separates the experimental system, tRNA state, codon dwell, and reported phenotype. Upward and downward arrows denote increases and decreases, respectively. The red rightward arrow contrasts tRNA availability in different experimental systems; arrows between amino acid names denote the direction of substitution. Filled and open circles in the NIH3T3 tRNA row denote relatively preserved and reduced aminoacylated-tRNA signal, respectively. The tRNA row distinguishes these relative measurements from availability inferred in cancer-cell experiments; conventional charging assays alone do not identify the attached amino acid. These qualitative summaries do not imply a common quantitative scale across studies; interpretation of the acute Leu phenotype as initiation-dominant is distinguished from the directly measured charging and dwell-time changes. None of these studies directly measured disomes, ribosome collisions, or ribosome-associated quality-control engagement. Abbreviations: BCAA, branched-chain amino acid; tRNA, transfer RNA; IARS1, cytoplasmic isoleucyl-tRNA synthetase 1; RQC, ribosome-associated quality control; CAR, pooled CAA/CAG anticodon isoacceptors (R = A or G); WAG, pooled AAG/UAG anticodon isoacceptors (W = A or U); I, inosine; Ψ, pseudouridine. Adapted from a figure created in BioRender. Kim, D. (2026). https://BioRender.com/ai2vcvx.

Table 2 summarizes the experimental systems and evidence boundaries, and Figure 2 compares the reported single-BCAA phenotypes. The strongest controlled comparison of the three BCAAs currently available is a six-hour deprivation study in mouse NIH3T3 fibroblasts [12]. It combined periodate-based tRNA charging assays with RNA sequencing (RNA-seq), ribosome profiling (Ribo-seq), polysome profiling, O-propargyl-puromycin incorporation, signaling measurements, and tandem mass tag proteomics. The study reported ribosome density as the Ribo-seq/RNA-seq ratio. Although this ratio is often termed translation efficiency, it measures relative ribosome occupancy per mRNA and does not directly quantify completed protein production; higher occupancy can also result from slower elongation. All deprivation conditions activated the ISR and reduced protein synthesis, although combined Leu/Ile deprivation (the study’s “double” condition) showed the weakest ISR activation and simultaneous Leu/Val/Ile deprivation (the “triple” condition) produced the largest reduction in nascent synthesis. The independent primary biological evidence for BCAA-specific translational phenotypes nevertheless remains narrow: Darnell et al. [5], Kok et al. [11], Worpenberg et al. [12], and Kochavi et al. [43]. Two subsequent computational studies reanalyzed data generated in the Worpenberg study rather than providing independent biological replication: Riboclette modeled the ribosome-footprint profiles [44], whereas a tensor-decomposition study integrated the transcriptome, translatome, and proteome layers [45].

### 3.1. Leucine Deprivation: Efficient Demand Control Can Dominate the Phenotype

Leu is both a protein substrate and a potent nutrient signal. Direct Leu sensing by Sestrin2 provides one molecular route to mTORC1 regulation [1,2], while the LARS1–RagD model provides a second, potentially coordinated route [36,37,38]. In the NIH3T3 comparison, Leu deprivation decreased mTORC1 outputs, activated the ISR, and reduced protein synthesis, while its codon-specific elongation signature was modest relative to Val deprivation [12]. The periodate-based assay nevertheless detected a substantial, group-selective reduction in aminoacylated tRNA signal—approximately 60% in the measured tRNA^Leu^(CAR) and tRNA^Leu^(WAG) groups relative to full-medium controls—while tRNA^Leu^(UAA) was largely spared; among Leu codons, only CUU showed a significant dwell-time increase [12]. This estimate describes a relative change in the aminoacylated tRNA signal, not a 60-percentage-point decrease in the absolute charged fraction. Reduced aminoacylated tRNA abundance therefore coexisted with limited codon-level pausing, consistent with demand control masking most codon-level effects (Section 2.2).

Darnell and colleagues used human HEK293T, HeLa, and HCT116 cells and likewise observed little Leu-codon pausing; by contrast, Arg limitation produced greater selective tRNA charging loss, codon pausing, and effects on protein output [5]. This comparison supports demand control as one explanation for modest Leu pausing but does not make it a universal property of Leu deprivation.

The key inference is not that Leu charging is irrelevant, but that initiation control can dominate the observable phenotype in some systems. Available studies span mouse NIH3T3 cells and human HEK293T, HeLa, HCT116, and several other cancer cell lines; none establishes a universal Leu response. Strong demand reduction may prevent large queues even when supply is limited. Leu limitation should therefore be characterized along two axes—nutrient signaling and decoding supply—rather than assigned categorically to one.

Longer Leu deprivation in selected human cancer cells produced a different pattern. After 48 h of Leu deprivation, MDA-MB-231, PC3, and MD55A3 cancer cells showed prominent UUA-biased ribosome occupancy, and +1 frameshift reporters were validated across MDA-MB-231, PC3, and A549 cells [43]. The abnormal products were detected only weakly in non-transformed human RPE1 and BJ cells, emphasizing cell-context dependence. tRNA^Leu^(UAA) overexpression mitigated the deprivation-induced reporter defect [43]. Thus, acute NIH3T3 deprivation spared tRNA^Leu^(UAA), whereas prolonged deprivation in selected cancer cells limited its availability, providing a direct example of system- and time-dependent isoacceptor behavior [12,43]. A convergent but distinct route operated during replication stress, in which Schlafen family member 11 (SLFN11)-dependent cleavage reduced tRNA^Leu^(UAA) availability and promoted UUA-biased frameshifting in SLFN11-positive cancer cells [43].

### 3.2. Valine Deprivation: Broad Loss of tRNA Charging and Multi-Codon Pausing

Val deprivation produced broad charging loss across the measured Val-tRNA isoacceptor groups and increased occupancy at all four Val codons in mouse NIH3T3 cells [12]. The limitation is therefore distributed across decoding routes rather than confined to one isoacceptor. This broad decrease refers to the tRNA groups resolved by the periodate-based reverse-transcription quantitative polymerase chain reaction (RT-qPCR) assay. Some measurements pool distinct anticodon isoacceptors, denoted by International Union of Pure and Applied Chemistry (IUPAC) ambiguity codes, and may also include unresolved isodecoders, which are distinct tRNA sequences sharing an anticodon. For example, the Leu CAR and WAG groups combine CAA/CAG and AAG/UAG anticodons, respectively. Charging decreased across all measured Val-tRNA groups, but individual tRNA species within a pooled group were not fully resolved [12].

Riboclette, a conditional deep-learning framework trained on the same Worpenberg ribosome-footprint profiles, assigned predictive importance to codon identity, local sequence context, and motif position, but it did not provide independent biological replication [44].

In the triple Leu/Val/Ile-deprivation condition, Worpenberg and colleagues proposed that strong early Val stalls could reduce the number of ribosomes reaching downstream Ile codons, thereby creating an apparent transcript-level 5′ bottleneck [12]. This was offered as a possible explanation for attenuation of downstream Ile-specific signals in the triple condition, not as a general property of Val deprivation. The interpretation is plausible but rests on density alone. Testing it requires position-controlled reporters and direct measurements of queued ribosomes, because monosome profiles cannot distinguish a long-lived isolated stall from a collision-prone queue.

Intervention experiments strengthen the supply–demand interpretation. Blocking elongation with cycloheximide during the final 30 min of deprivation restored tRNA charging. During simultaneous Leu, Val, and Ile deprivation, combined proteasome and autophagy inhibition intensified Val-tRNA charging loss without producing comparable Ile-tRNA deacylation [12]. Ongoing translation demand and intracellular amino acid recycling therefore help determine tRNA charging. The recycling experiment had only two replicates, so it should be weighed accordingly.

A 2026 tensor-decomposition analysis of the same RNA-seq, Ribo-seq, and proteomic dataset identified clusters with increased transcript and ribosome-footprint signals but reduced protein abundance, a pattern interpreted as consistent with ribosome stacking [45]. Because it reused the same data and did not directly measure collided ribosomes, it supports cross-omic translational discordance but not independent evidence of collision.

### 3.3. Isoleucine Deprivation: Isoacceptor Selectivity and a Fidelity Bypass

In mouse NIH3T3 cells, Ile deprivation showed a more selective charging phenotype. The adenosine deaminase acting on tRNA (ADAT2/3) heterodimer converts adenosine 34 to inosine (I34), so the mature IAU form decodes AUU and AUC strongly and AUA more weakly [46]. Charging of the genomic tRNA^Ile^(AAU) group fell, whereas the pseudouridine (Ψ)-containing UAU/ΨAΨ route that redundantly supports AUA decoding was relatively preserved. Accordingly, AUU and AUC displayed stronger pausing than AUA [12]. AUA showed no comparable pause even though IAU can decode it, consistent with the preserved AUA-supporting route remaining sufficient to meet demand.

A separate study examined four control human fibroblast lines and two patient-derived human fibroblast lines carrying the same compound-heterozygous *IARS1* genotype. Purified wild-type cytoplasmic isoleucyl-tRNA synthetase 1 (IARS1) directly generated Val-tRNA^Ile^ in vitro using in vitro-transcribed tRNA^Ile^. In cells, the evidence was indirect: *IARS1*-genotype-dependent Ile-to-Val peptides were detected from an exogenous reporter during Ile restriction [11]. These evidence levels should not be merged, and neither experiment directly connects the substitution to the NIH3T3 pause pattern.

The cellular mass-spectrometric Ile-to-Val evidence was confined to two Ile-containing peptides from an exogenous green fluorescent protein (GFP)–catenin alpha 1 (CTNNA1) reporter; no endogenous-protein substitution was demonstrated. Val supplementation modestly improved a 15-Ile repeat reporter in patient cells and improved proliferation in control but not IARS1-deficient cells. The Val concentration was 2330 µM—approximately ten times the cited plasma concentration and 5.2 times the standard medium concentration. Although Val improved reporter output and growth, it did not further increase the Ile-to-Val signal in the two measured CTNNA1 peptides [11]. Endogenous proteome-wide substitution rates and their quantitative relation to functional rescue therefore remain unknown.

The same work detected Ile-to-Met substitution in both healthy and IARS1-deficient cells, while Met supplementation did not rescue growth [11]. The patient-derived cells retained IARS1 activity, so this comparison alone does not establish complete IARS1 independence or identify the decoding route. Near-cognate decoding and alternative tRNA misacylation remain possible explanations; stress-associated methionine misacylation is discussed in Section 5. The Ile-to-Met signal is therefore treated here as a substitution of unresolved mechanism, rather than evidence establishing ribosomal misdecoding.

Keeping translation going and rescuing biological function are also different outcomes. A reporter signal or short-term growth benefit can increase even if a subset of endogenous proteins loses activity, stability, or localization. The long-term balance among rescued synthesis, proteotoxic burden, and selective degradation has not yet been defined.

### 3.4. Combined BCAA Deprivation Is Not a Simple Sum

Combined deprivation changes both supply and ribosome load (Table 2). In the mouse NIH3T3 study, combined Leu and Ile deprivation (the study’s “double” condition) produced the strongest inhibition of selected mTORC1 readouts. It also gave the weakest ISR activation and only mild codon-specific dwell-time changes; among BCAA codons, only AUU increased significantly. By contrast, simultaneous Leu, Val, and Ile deprivation (the “triple” condition) caused the greatest reduction in O-propargyl-puromycin incorporation, largely retained the Val-dominant elongation signature, and showed a 5′ shift in ribosome density [12]. These observations argue against predicting a combined condition by adding the effects of three individual amino acid deprivations. A strong initiation response to one component can reduce the ribosome demand that reveals another component’s elongation defect.

## 4. Ribosome State Can Feed Back to GCN2 and Initiation Control

**Figure 3:**
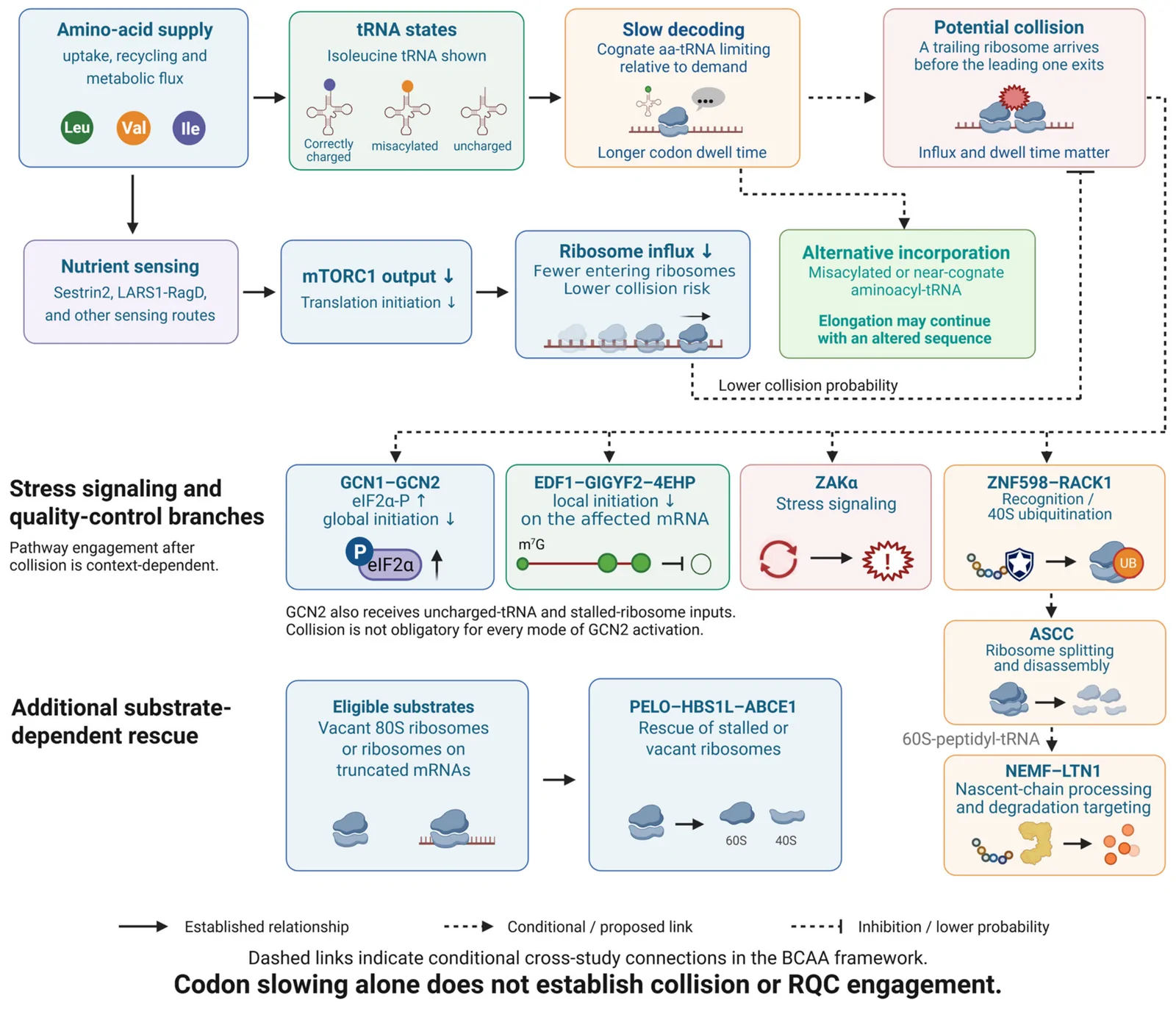
Feedback architecture linking amino acid supply, aminoacyl-tRNA state, ribosome influx, potential collision, and surveillance. Amino acid uptake, recycling, and metabolic flux shape the correctly charged, misacylated, and uncharged tRNA pools. Reduced cognate charging can slow decoding at a codon, but collision occurs only conditionally when effective ribosome influx allows a trailing ribosome to reach the delayed leading ribosome. Misacylated or near-cognate decoding can instead sustain elongation while altering protein sequence and is shown as a route distinct from collision formation. Sestrin2, LARS1–RagD, and other nutrient-sensing routes regulate mTORC1 output and thereby alter translation initiation and effective ribosome influx [1,2,36,37,38]. Uncharged tRNA, the stalled A-site state, and GCN1–ribosome contacts can contribute to GCN2 activation in a stress- and system-dependent manner [3,47,48,49,50,51,52,53,54,55,56]. Potential ribosome collisions can engage GCN1–GCN2–eIF2α global initiation feedback [53,55,56], EDF1–GIGYF2–4EHP transcript-local initiation feedback [13,14], and ZAKα-dependent stress signaling [7,58,59,60]. ZNF598–RACK1-mediated collision recognition and 40S ubiquitination [8,61,62], ASCC-dependent splitting and disassembly [63,64], NEMF–LTN1-mediated nascent-chain triage [9,10,16,17,18,65,66], and PELO–HBS1L–ABCE1-mediated rescue of stalled or vacant ribosomes [67,68] are interacting but separable branches rather than a single obligatory pathway. Solid arrows indicate established regulatory or decoding relationships; dashed arrows indicate context-dependent connections or links not directly demonstrated during BCAA deprivation. Blunt-ended lines denote inhibition or reduced probability; ↑ and ↓ denote increases and decreases, respectively. Abbreviations: aa-tRNA, aminoacyl-tRNA; BCAA, branched-chain amino acid; LARS1, leucyl-tRNA synthetase 1; RagD, Ras-related GTP-binding protein D; mTORC1, mechanistic target of rapamycin complex 1; GCN1, general control nonderepressible 1; GCN2, general control nonderepressible 2; eIF2α, eukaryotic initiation factor 2α; eIF2α-P, phosphorylated eIF2α; EDF1, endothelial differentiation-related factor 1; GIGYF2, GRB10-interacting GYF protein 2; 4EHP, eIF4E homologous protein; ZNF598, zinc finger protein 598; RACK1, receptor for activated C kinase 1; ASCC, activating signal cointegrator 1 complex; ZAKα, α isoform of mitogen-activated protein kinase kinase kinase 20; NEMF, nuclear export mediator factor; LTN1, listerin E3 ubiquitin protein ligase 1; PELO, pelota mRNA surveillance and ribosome rescue factor; HBS1L, HBS1-like translational GTPase; ABCE1, ATP-binding cassette subfamily E member 1. Adapted from a figure created in BioRender. Kim, D. (2026). https://BioRender.com/ai2vcvx.

The BCAA-specific phenotypes described above raise a common mechanistic question: can a local decoding delay feed back to global initiation control? Figure 3 outlines the signaling and surveillance branches considered in this and the following sections. The classical model places uncharged tRNA upstream of GCN2. Biochemical work on *S. cerevisiae* Gcn2 showed that uncharged tRNA binds the histidyl-tRNA synthetase-like region and relieves kinase autoinhibition [47]. Subsequent mammalian and structural studies identified the ribosomal P-stalk and general control nonderepressible 1 (GCN1) as additional inputs, and cryo-electron microscopy showed *S. cerevisiae* Gcn1 spanning leading and colliding ribosomes [48,49,50,51,52].

The relative weight of these inputs varies by stress and system. In human HEK293T cells, activation of GCN2 by some elongation inhibitors required mitogen-activated protein kinase kinase kinase 20 (MAP3K20/ZAK), whose long isoform, ZAKα, functions as a collision sensor; the knockout used in that study was not isoform-specific. By contrast, tRNA deacylation in HEK293T cells and ultraviolet stress in HEK293T cells and human NTERT keratinocytes retained a more direct uncharged-tRNA component [3]. In a reconstituted in vitro translation system, collision-dependent GCN1 recruitment and GCN2–ribosome contacts initiated signaling, and GCN2 remained stably associated with collided ribosomes [53]. How much this collision-coupled route contributes, relative to free uncharged tRNA, under physiological amino acid limitation remains unresolved.

Experiments in *S. cerevisiae* add further boundary conditions. Tethered P1/P2 heterodimers were dispensable for Gcn2 activation during histidine starvation and during sulfometuron-methyl-induced combined Ile/Val starvation, although their requirements differed under other stresses [54]. Thus, the latter experiment provides a BCAA-relevant condition but is not starvation for a single amino acid. Multiprotein bridging factor 1 (Mbf1) was required for robust Gcn2 activation on collided ribosomes in another yeast system [55]. Together, these results indicate that free uncharged tRNA and the A-site decoding state are not interchangeable inputs [3,47,53]. Contributions from the P-stalk [48,49,54], GCN1–ribosome contacts [50,51,52,53], and collision-associated factors [55,56] vary by stress and system; they do not form a fixed sequence of steps. The structural logic, signaling networks, and disease links of GCN2 have been reviewed comprehensively [57].

For BCAA deprivation specifically, the feedback model remains incomplete. Worpenberg and colleagues documented codon occupancy, ISR activation, mTORC1 outputs, and protein synthesis, but did not directly map disomes or test GCN1 or GCN2 recruitment to queues arising during BCAA deprivation [12]. The proposal that BCAA-specific pauses feed back through collisions is therefore mechanistically plausible and testable, but not yet demonstrated. Disome profiling, selective perturbation of GCN1 interfaces, and matched tRNA charging measurements will be required. Figure 3 integrates established and provisional feedback connections while preserving their evidence boundaries.

## 5. Translational Fidelity Under Amino Acid Limitation

Reduced charging can affect more than ribosome speed: under some conditions, it can also alter which amino acid is incorporated. aaRSs must select the correct amino acid and the correct tRNA while rejecting near-cognate substrates. Fersht’s biochemical analysis of Val rejection by *E. coli* isoleucyl-tRNA synthetase established the importance of editing in achieving fidelity when substrates are structurally similar [69]. Limitation disturbs this balance in two ways. It slows the production of correctly charged tRNA, and it may give a non-cognate amino acid or a near-cognate tRNA more opportunity to enter decoding. The resulting substitution frequency depends on enzyme editing, substrate ratios, tRNA competition, and ribosome kinetics, not on amino acid concentration alone. A recent review places tRNA identity, charging, editing, delivery, and codon–anticodon selection within a common framework for understanding mistranslation [70].

Three mechanisms that alter protein sequence must be separated: tRNA misacylation, near-cognate misdecoding, and frameshifting. Table 3 summarizes the BCAA-linked evidence for these routes; because the route underlying the Ile-to-Met signal is undetermined, that case is listed separately as an unresolved substitution rather than assigned to a single mechanism. Misacylation occurs when a tRNA with a cognate anticodon carries the wrong amino acid, as in Val-tRNA^Ile^ decoding an Ile codon. Near-cognate misdecoding occurs when a tRNA is accepted through imperfect codon–anticodon pairing. The Ile-to-Met signal during Ile deprivation does not by itself distinguish this route from alternative tRNA misacylation. Frameshifting changes the reading frame itself, as in UUA-dependent +1 events during Leu deprivation [43]. These routes can all alter protein sequence but have different causes, kinetics, and validation requirements.

Mistranslation is not intrinsically adaptive or deleterious. In *E. coli*, ambiguous decoding can be tolerated at surprisingly high levels when chaperone and degradation systems buffer the proteome, although this buffering has an energetic cost [71]. Oxidative stress provides examples in both directions. Human HeLa cells can increase methionine misacylation on non-methionyl tRNAs during innate immune or chemical oxidative stress, a response proposed to protect proteins through additional methionine residues [72]. This establishes an alternative route to Met incorporation, although it does not identify the mechanism of the Ile-to-Met signal during Ile deprivation. In *E. coli*, oxidation of the editing site of threonyl-tRNA synthetase produces Ser-for-Thr errors, protein misfolding, and impaired growth [73]. These studies show that the phenotype depends on which substitutions occur, in which proteins, and on the available proteostasis capacity.

Under the operational definition in Table 1, adaptive translation during BCAA deprivation remains a hypothesis. Confirming it requires evidence that a defined altered protein product improves cellular fitness under the relevant stress, not merely the detection of a stress-associated error [74]. Ile-to-Val substitution during Ile deprivation partially meets this test because reporter output and proliferation improved in an IARS1-dependent manner [11]. However, IARS1 dependence is consistent with, but does not establish, causal mediation by Ile-to-Val substitution; improved canonical Ile charging or other IARS1-dependent effects remain alternative explanations. Limited peptide coverage, unequal assay conditions, and the absence of an additional Ile-to-Val increase after Val supplementation leave the endogenous sites, substitution frequencies, and responsible functional products unresolved.

Proteome-wide detection of translation-associated amino acid substitutions is technically difficult. Search strategies can confuse true substitutions with sample-preparation artifacts, post-translational modifications, sequence variants, or false peptide-spectrum matches [75,76]. Nevertheless, stress-dependent Trp-to-Phe and Arg-to-Cys substitutions demonstrate that amino acid limitation can leave reproducible signatures in mammalian proteomes [77,78]. Near-cognate decoding kinetics and codon context further complicate attribution [39]. Rigorous studies should combine isotope-aware targeted mass spectrometry, synthetic peptide validation, genomic controls, and measurements of the responsible aaRS/tRNA route. Comparative evaluations of misincorporation proteomics workflows further clarify which detection strategies are reliable [79].

Changes in translational fidelity may be especially consequential in long-lived and postmitotic cells. Reviews and primary studies of aaRS defects and neurological disease indicate that modest errors in tRNA charging, translation, or ISR regulation can produce selective neuronal vulnerability [80,81,82,83]. More broadly, translation is increasingly recognized as a constraint on proteostasis during aging [84]. In vivo reporter analysis further found organ-dependent increases in stop-codon readthrough with age in mouse muscle and brain, but not liver [85]. This readout is mechanistically distinct from BCAA-deprivation-associated amino acid substitution, and neither observation establishes that dietary BCAA restriction causes neurological disease. Together, they show why a short-term gain in translation should not be read as a favorable long-term outcome without measurements of protein function, tissue context, and age. Whether a fidelity change remains a local sequence alteration or contributes to a broader proteostasis problem depends on both the affected protein and the fate of any slowed ribosome.

## 6. From Ribosome Slowing to Collision, RQC, and Cell Fate

**Figure 4:**
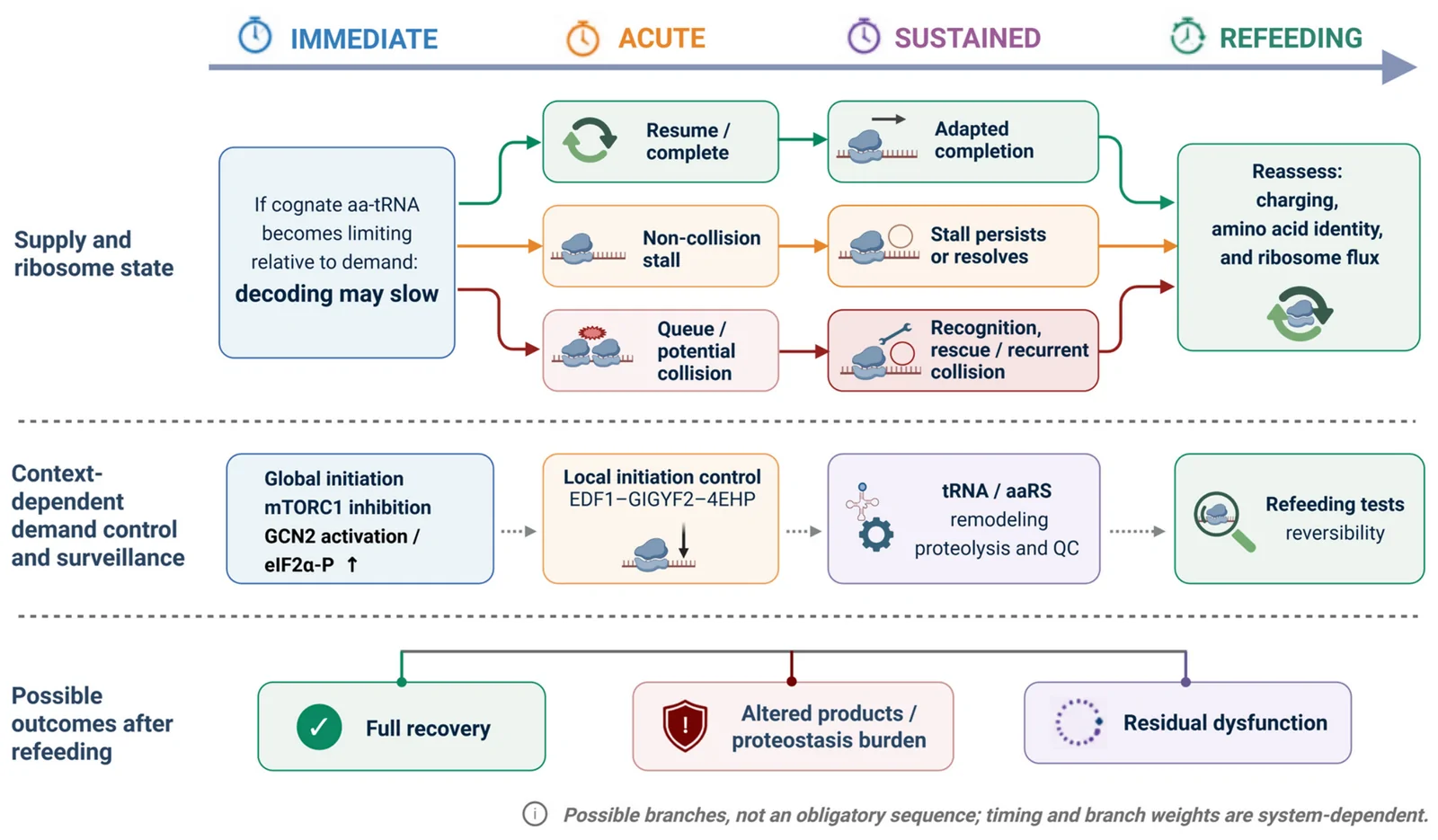
Time-dependent branching between reversible adaptation and persistent dysfunction during BCAA limitation and refeeding. When deprivation reduces the availability of correctly aminoacylated cognate tRNA relative to demand, decoding can slow; this is the immediate phase. During the acute phase, an engaged ribosome may resume and complete translation, remain in a non-collision stall, or develop a queue and potential collision. Under sustained limitation, these three branches may lead respectively to adapted completion, persistence or resolution of a stall, or recognition followed by rescue or recurrent collision. Global demand control through mTORC1 inhibition and GCN2–eIF2α activation, transcript-local initiation control through EDF1–GIGYF2–4EHP, and remodeling of tRNA, aminoacyl-tRNA synthetases, proteolysis, and quality-control capacity act as context-dependent modifiers rather than an obligatory sequence. Refeeding permits reassessment of charging, attached amino acid identity, and ribosome flux and can reveal full recovery, persistent altered products or proteostasis burden, or residual dysfunction. The branches and time windows are conceptual; their timing and relative weights depend on the cell type, transcript, deprivation condition, and duration. The top arrow indicates time progression. Green, orange, and red arrows trace possible completion, non-collision stall, and collision-related branches, respectively. Gray dotted arrows link con-text-dependent modifiers rather than obligatory steps; ↑ denotes an increase. Abbreviations: aa-tRNA, aminoacyl-tRNA; BCAA, branched-chain amino acid; mTORC1, mechanistic target of rapamycin complex 1; GCN2, general control nonderepressible 2; eIF2α, eukaryotic initiation factor 2α; eIF2α-P, phosphorylated eIF2α; EDF1, endothelial differentiation-related factor 1; GIGYF2, GRB10-interacting GYF protein 2; 4EHP, eIF4E homologous protein; tRNA, transfer RNA; aaRS, aminoacyl-tRNA synthetase; QC, quality control. Adapted from a figure created in BioRender. Kim, D. (2026). https://BioRender.com/ai2vcvx.

The operational criteria in Table 1 distinguish slowing, collision, and downstream quality-control engagement. Their application requires attention to both ribosome arrival rates and the duration and resolution of stalls. Figure 4 previews how these states can resolve or persist across the immediate, acute, sustained, and refeeding phases.

An illustrative timescale comparison sharpens this distinction. Measurements in mouse organs yielded elongation rates of approximately 6.8, 5.0, and 4.3 amino acids per second in liver, kidney, and skeletal muscle, corresponding to mean codon-passage times of about 0.15–0.23 s [86]. Low-density single-mRNA measurements in human HeLa cells reported slower elongation of approximately 2–4.5 amino acids per second and initiation frequencies of roughly one to two events per minute, corresponding to passage times of about 0.22–0.5 s and initiation intervals of tens of seconds [41]. Because these estimates were obtained in different systems and by different methods, they should not be treated as interchangeable. The single-mRNA work [41] and the BCAA-deprivation study [12] use distinct datasets; shared investigators do not make those datasets identical, although replication across laboratories remains valuable. A several-fold increase in mean dwell time nevertheless need not make collision the dominant outcome. Collisions become more likely on highly loaded transcripts, during long-tailed persistent stalls, or across multiple pause sites. These estimates are illustrative rather than universal; transcript-specific ribosome influx and the full stall-duration distribution require calibrated monosome and disome profiling [15,40].

Pharmacologic aminoacyl-tRNA synthetase inhibition provides a direct counterexample to treating aminoacylation failure as synonymous with collision. In experiments using human cell lines, including U-2 OS, collided ribosomes were cleared in a ZNF598-dependent manner, whereas uncollided stalls persisted for more than 16 h despite ISR activation [87]. The associated block in ribonucleoprotein granule assembly was also observed during amino acid deprivation, although BCAA-specific conditions were not tested. Stall persistence, collision formation, and collision clearance are therefore separable variables.

Disome and trisome profiling in *S. cerevisiae* shows that endogenous collisions occur at defined transcript features and do not all produce the same outcome [15]. Mechanistic studies further separate collision recognition and ubiquitination, ribosome splitting and disassembly, post-splitting processing, and rescue into interacting but distinct response branches [61,62,63,64,65,66,67,68]. Zinc finger protein 598 (ZNF598) and receptor for activated C kinase 1 (RACK1) recognize collision-associated ribosome states and promote regulatory 40S ubiquitination [8,61,62], whereas activating signal cointegrator 1 complex (ASCC) factors participate in ribosome splitting and disassembly [63,64]. Notably, canonical collision geometry is not an absolute prerequisite for ZNF598-dependent ubiquitination and ASCC-mediated disassembly in all experimental settings [64]. Collision formation, recognition, ubiquitination, and ribosome disassembly should therefore be measured as separable events rather than inferred as consecutive steps in a single linear pathway.

After splitting, nuclear export mediator factor (NEMF) and listerin E3 ubiquitin protein ligase 1 (LTN1) process the separated 60S–nascent-chain complex [9,10,16,17,18,65,66]. Pelota mRNA surveillance and ribosome rescue factor (PELO), HBS1-like translational GTPase (HBS1L), and ATP-binding cassette subfamily E member 1 (ABCE1) rescue stalled or vacant 80S complexes [67,68]. These factors should not be presented as consecutive steps in one universal linear pathway.

Collision also provides a signaling platform. ZAKα can activate p38 and c-Jun N-terminal kinase (JNK) branches of the ribotoxic stress response, GCN2–eIF2α can lower global initiation, and EDF1–GIGYF2–4EHP can reduce initiation locally on the same mRNA [7,13,14,58,59,60]. GCN1 also engages an E3-ligase network that degrades translation factors on stalled ribosomes [88]. A 2026 eLife Reviewed Preprint proposed that starvation-induced ring finger protein 25 (RNF25) and GCN2 compete for GCN1 [89]. Direct evidence for this competition, and for collision resolution, remains incomplete, and the eLife public assessment likewise judged the evidence for these key mechanistic claims incomplete. A peer-reviewed study independently showed that RNF25 promotes tolerance of azacytidine-induced mRNA damage by ubiquitinating ribosomal eS31 and curbing ISR activation [90]. This RNA-damage mechanism has not yet been established as a response to BCAA deprivation. The collision-response network therefore acts as a triage system spanning stress signaling, local demand control, rescue, surveillance, and nascent-chain degradation—not simply disposal [16,17,18,68]. Dysregulation of these ribosome quality-control pathways contributes to a range of human diseases [91].

For BCAA deprivation, direct engagement of these branches remains a major evidence gap. The observed Val- and Ile-codon density increases identify candidate slow sites but do not demonstrate disomes, ubiquitinated ribosomes, nascent-chain degradation, or mRNA cleavage [12]. Reduced downstream density can arise from lower initiation, an early bottleneck, ribosome drop-off, or altered mRNA stability. Because codon identity itself affects mRNA stability in human cells [92] and *S. cerevisiae* [93], this ambiguity matters. Claims should therefore match the measured level of the evidence ladder.

Time is an explicit variable in the surveillance response. During the acute phase, over minutes to hours, inhibition of initiation can rapidly lower ribosome influx, while amino acid recycling and residual charged tRNA permit many engaged ribosomes to finish translation. Measurements in this window should distinguish the order of tRNA deacylation, codon pausing, disome formation, and ISR or RSR activation rather than treating a single endpoint as the complete response.

During sustained restriction, the system itself changes: transcriptional adaptation, proteolysis, tRNA abundance and modification, aaRS expression, and chaperone and proteasome capacity can all reshape the same initial bottleneck. Persistent queues or fidelity changes may then consume quality-control capacity and generate effects that are absent during a short pulse. Oxidant-induced ribosome damage and ZAKα signaling provide a precedent for how chronic ribosome impairment can contribute to metabolic decline [94]. The broader impact of oxidative stress on ribosome function has been reviewed [95]. Refeeding and recovery experiments are essential to distinguish a reversible acute adaptation from a proteostasis cost that persists after amino acid supply is restored. Whether chronic dietary BCAA restriction engages a comparable ribosome stress axis in specific tissues remains unknown.

## 7. Two-Layer Kinetic Framework for Amino Acid Resource Allocation in Translational Control

### 7.1. Two-Layer Kinetic Structure

**Figure 5:**
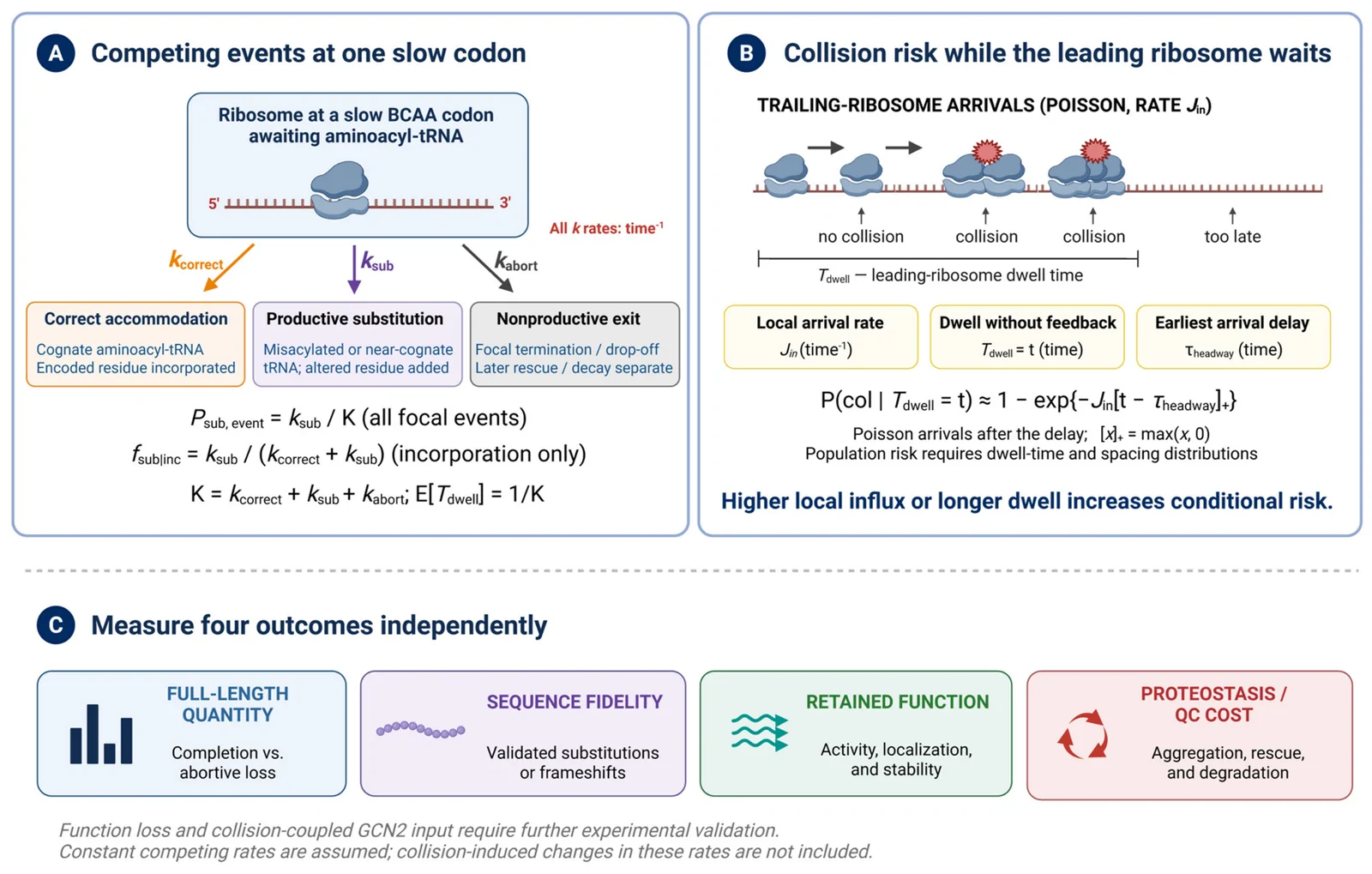
Two-layer kinetic framework with distinct event probabilities, product fractions, and conditional collision risk. (**A**) Correct accommodation (*k*_correct_), substitution (*k*_sub_), and focal nonproductive exit (*k*_abort_) are effective, approximately constant hazards (time^−1^). *P*_sub,event_ includes abortive events in its denominator, whereas *f*_sub|inc_ is conditional on amino acid incorporation. Correspondence to measured peptide fractions additionally requires comparable downstream completion and survival and correction for analytical response. With *K* = *k*_correct_ + *k*_sub_ + *k*_abort_, E[*T*_dwell_] = 1/*K* before collision-induced kinetic changes. (**B**) *J*_in_ is a local trailer-arrival rate (time^−1^), *T*_dwell_ is the dwell duration without collision-induced feedback, and *τ*_headway_ is an earliest-arrival delay set by initial spacing and upstream motion. Assuming no arrivals before this delay and Poisson arrivals thereafter, P(col|*T*_dwell_ = *t*) ≈ 1 − exp{−*J*_in_[*t* − *τ*_headway_]_+_}, with [*x*]_+_ = max(*x*, 0). This is a conditional approximation; population estimates require dwell-time and spacing distributions. Exclusion, existing queues, and feedback can invalidate the approximation. After collision, the ribosome may resume elongation or engage signaling, rescue, or quality control. (**C**) Full-length quantity, sequence fidelity, retained function, and proteostasis cost are separate outcomes. Functional impairment and collision-coupled GCN2 activation are biological hypotheses to test, not necessary consequences of the equations. In (**A**), orange, purple, and gray arrows indicate correct accommodation, substitution, and non-productive exit, respectively. In (**B**), horizontal arrows indicate ribosome movement; vertical arrows identify the illustrated arrival outcomes. Abbreviations: BCAA, branched-chain amino acid; tRNA, transfer RNA; QC, quality control. Adapted from a figure created in BioRender. Kim, D. (2026). https://BioRender.com/ai2vcvx.

The evidence reviewed above motivates a two-layer kinetic framework—heuristic rather than fitted—that combines established mechanisms with explicit modeling assumptions and additional biological hypotheses (Figure 5). It separates resolution of a slow A-site decoding event from the concurrent arrival of a trailing ribosome. The first layer describes competing, approximately constant hazards during one focal decoding event. Amino acid flux sets the pools of correctly charged, misacylated, and uncharged tRNAs. Local tRNA competition sets the effective rates of correct accommodation (*k*_correct_), productive substitution through misacylation or near-cognate decoding (*k*_sub_), and nonproductive exit from the focal event (*k*_abort_). All three rates have units of time^−1^. The probability per focal event of resolution through substitution is given by Equation (1):

*P*_sub,event_ = *k*_sub_/(*k*_correct_ + *k*_sub_ + *k*_abort_) (1)

Here, *k*_abort_ pools nonproductive exits at the focal codon, including premature termination or ribosome drop-off represented at this level of abstraction. It does not identify a particular rescue pathway, and subsequent splitting and degradation require separate states. Conditional on amino acid incorporation at that codon, the substitution fraction is instead given by Equation (2):

*f*_sub|inc_ = *k*_sub_/(*k*_correct_ + *k*_sub_) (2)

This conditional fraction can approximate the substituted/(canonical + substituted) molar peptide fraction only if subsequent completion and survival probabilities are comparable and analytical response differences are corrected. Otherwise, protein completion, selective turnover, and peptide detectability bias the measured ratio. Defining *K* = *k*_correct_ + *k*_sub_ + *k*_abort_, the same constant-hazard model gives E[*T*_dwell_] = 1/*K* for the focal waiting state before collision-induced changes in kinetics; mean dwell time and exit rates are therefore coupled.

Collision formation can occur while the focal ribosome is still waiting. Let *J*_in_ denote an effective local arrival rate of trailing ribosomes upstream of that site, measured as ribosomes per unit time (units of time^−1^), rather than equating it with initiation at the start codon. Let *T*_dwell_ be the dwell duration in the absence of collision-induced feedback and *τ*_headway_ an effective earliest-arrival delay determined by the initial ribosome spacing and upstream motion; both have units of time. As an illustrative shifted-arrival approximation, assume no trailer reaches the site before *τ*_headway_ and arrivals thereafter follow a Poisson process with rate *J*_in_. Conditional on *T*_dwell_ = *t*, the probability of at least one collision is given by Equation (3):

P(col|*T*_dwell_ = *t*) ≈ 1 − exp{−*J*_in_[*t* − *τ*_headway_]_+_} (3)

Here, [*x*]_+_ = max(*x*, 0). The exponent is dimensionless because an arrival rate multiplies a waiting time. With spacing and upstream motion fixed, higher local influx or a longer dwell increases collision probability; reduced initiation can lower local influx without restoring correct charging. Population-level collision probability requires averaging over the dwell-time and spacing distributions: substituting a mean dwell time generally gives a different result. Ribosome exclusion, pre-existing queues, and feedback can violate the shifted-Poisson approximation. Once collision occurs, the leading ribosome may resume correct or substituted elongation, terminate, be rescued, or undergo quality-control processing, and its effective rates can change. The two layers thus describe interacting processes rather than mutually exclusive final fates.

Sequence fidelity and retained function are distinct outputs. A conservative Ile-to-Val substitution at a permissive site may lower fidelity while preserving activity, stability, and localization, whereas a single substitution at an active site, interaction interface, or hydrophobic core may abolish function [11]. Protein amount, validated substitution frequency, and retained activity should therefore be measured separately, because the same global error rate can mean very different things for different proteins. Work in a *Neurospora crassa* cell-free translation system shows that local elongation rate can shape co-translational folding [96]. This provides a mechanistic basis for testing whether BCAA-associated pauses affect folding in mammalian cells, independently of any sequence change.

This abstraction is site-, transcript-, cell-type-, and time-dependent. Focal premature termination and drop-off are pooled in *k*_abort_, but their molecular mechanisms and subsequent rescue or degradation require additional states. Frameshifting, readthrough, transcript-local initiation inhibition, and mRNA loss are outside the minimal equations. These equations organize dependencies and measurement requirements; they do not describe universal rate constants or a regulated cellular choice.

These effective parameters are not identifiable from any single steady-state measurement. A ribosome-density profile does not reveal whether a ribosome resumes correctly, substitutes, aborts, or is reached by another ribosome. A disome signal combines collision formation and resolution, while full-length protein integrates initiation, elongation, termination, mRNA decay, nascent-chain degradation, and post-translational turnover. Estimation therefore requires matched time courses of initiation load, isoacceptor charging, mono- and disome occupancy, nascent-chain completion, validated substitution peptides, and degradation during deprivation and refeeding. Orthogonal perturbations should vary initiation at matched charging, charging at comparable load, or IARS1 at matched Ile and Val supply.

### 7.2. Testable Predictions and Mechanistic Hypotheses

The framework defines two traffic predictions under the stated assumptions and two additional biological hypotheses. The latter concern protein function and GCN2 input and are not mathematical consequences of the competing-hazard equations. Each requires matched BCAA-deprivation conditions and an explicit scope for interpretation.

Prediction 1—codon placement and queue propagation: moving a Val- or Ile-sensitive codon cluster toward the 5′ end of an otherwise identical reporter should alter the position of the upstream queue and the downstream ribosome-density profile. A sufficiently slow bottleneck can reduce steady-state ribosome flux and protein output. However, moving an otherwise equivalent bottleneck toward the 5′ end does not necessarily cause an additional reduction in output. In the absence of ribosome loss, steady-state flux is conserved along an individual transcript. A stronger 5′-position effect on output is predicted only if the queue reaches the initiation region, triggers transcript-local initiation inhibition, promotes ribosome drop-off, or accelerates mRNA decay. The proposed position-dependent output effect is unsupported if, in a regime with a verified bottleneck and sufficient ribosome load, matched reporters show no corresponding changes in queue propagation, initiation, mRNA half-life, or full-length output.

Prediction 2—initiation load and local influx: for *t* > *τ*_headway_, Equation (3) predicts higher conditional first-collision risk with increasing *J*_in_ at fixed *t* and *τ*_headway_. In experiments, initiation changes may alter local influx and ribosome spacing together, depending on upstream elongation and the traffic regime. Their joint effect should therefore be evaluated from measured influx, dwell-time, and spacing distributions. Tests should compare BCAA-deprivation conditions with comparable availability of correctly charged cognate tRNA and use a prespecified, nonsaturated range in which the model predicts a detectable change. Collision formation must be distinguished from disome abundance, which also depends on resolution and clearance. An unchanged formation probability across such a verified range would challenge the joint prediction under those conditions; unchanged steady-state disome abundance alone would not.

**Hypothesis 1.**  *Quantity versus function: in a prespecified panel of endogenous proteins with independently established sensitivity to Ile-to-Val substitution, Val supplementation during Ile deprivation may restore full-length abundance more than specific activity. At matched sites, measure full-length abundance normalized to mRNA, calibrated molar substitution fraction (substituted peptide divided by canonical plus substituted peptide), and specific activity normalized to full-length protein. A disproportionate loss of specific activity associated with validated substitution would support this protein-specific hypothesis. IARS1 perturbations must account for residual canonical aminoacylation, baseline fitness, and effects unrelated to substitution. Equivalent recovery of amount and activity within a prespecified margin would oppose the hypothesis for that panel, while remaining compatible with the broader framework and function-preserving substitutions at permissive sites.*

**Hypothesis 2.**  *Collision-coupled GCN2 input: early GCN2 activation during BCAA deprivation may contain a collision-dependent component. Disrupting a GCN1–GCN2 interface alone tests pathway dependence, not selective dependence on collision. Tests should compare collision-prone and collision-low conditions with charging and local influx measured, and, where feasible, use separation-of-function perturbations that impair collision-coupled signaling while preserving other GCN2 responses. A collision-selective effect after these controls would support the hypothesis. Failure to detect such an effect would limit the inferred contribution in that system, rather than excluding all GCN1- or tRNA-dependent GCN2 activation.*

## 8. Physiological and Nutritional Relevance

The two-layer kinetic framework in Section 7 and Figure 5 defines testable cellular mechanisms, but nutritional relevance requires an additional step: determining whether dietary restriction creates the same ribosomal limitation in tissues. Complete removal of an amino acid from culture medium is a useful perturbation but differs from dietary restriction. Feeding–fasting cycles, proteolysis, tissue exchange, uptake, and catabolism buffer circulating supply, and brain, liver, muscle, immune cells, and tumors can experience different degrees and durations of limitation [86,97,98,99]. In sarcopenic C57BL/6 mice and senescence-accelerated mouse prone 8 (SAMP8) mice, circulating BCAAs were higher, whereas skeletal-muscle BCAAs and muscle expression of L-type amino acid transporter 1 (LAT1) and branched-chain aminotransferase 2 (BCAT2) were lower than in young controls [100]. Plasma abundance therefore need not report tissue access or handling; that study did not measure tRNA charging.

The operational definition of functional deficiency in Table 1 also applies to tissues: detectable amino acid does not exclude a supply–demand mismatch if cognate tRNA charging falls. Metabolomics and flux measurements describe supply, use, and recycling; charging and ribosome profiling test functional limitation at decoding. GCN2–eIF2α–ATF4 activation, lower mTORC1 output, and reduced bulk synthesis are supportive but not specific. Codon dwell time and charging have been co-measured in nutrient-stressed mouse liver, but such tissue-level evidence remains limited [99].

BCAAs are especially embedded in whole-body physiology. Skeletal muscle is a major site of BCAA transamination in mammals [21,22]. Human nutritional studies and reviews show that Leu-rich nutrition can stimulate mTORC1 and muscle protein synthesis, although anabolic resistance, immobilization, age, and total dietary context modify the outcome [101,102,103,104]. The catabolic enzymes branched-chain aminotransferase (BCAT) and branched-chain α-keto acid dehydrogenase (BCKD) that govern this flux have been reviewed [105]. Interventions that change one BCAA also alter the balance among the three and can influence amino acid transport and catabolic flux. A dietary phenotype cannot therefore be assigned to one ribosomal mechanism without measuring the relevant tissue aminoacyl-tRNA pools.

Restriction of individual BCAAs produces distinct metabolic and aging phenotypes in mice. Experiments in C57BL/6J mice identified distinct contributions of Ile and Val to adverse metabolic effects of a BCAA-rich diet [106]. Lifelong BCAA restriction in C57BL/6J mice had sex-specific effects on frailty and lifespan [107]. Ile restriction improves metabolic health and lifespan in genetically heterogeneous UM-HET3 mice, with effects differing between males and females [108]. Protein or Ile restriction initiated at 20 months of age alters aging signatures in male and female C57BL/6J NIA mice [109]. Lifelong Val restriction in C57BL/6J mice improves aspects of healthspan in both sexes and male median lifespan [110]. Individual BCAA restrictions also have distinct effects in male and female 3xTg mouse models of Alzheimer’s disease [111]. The three BCAAs are therefore not nutritionally interchangeable [105,112]. These strain-, sex-, age-, and tissue-dependent dietary studies do not directly demonstrate codon-specific slowing, substitution, collision, or RQC; instead, they motivate tissue-matched tests rather than confirm the cell-culture model.

Tumor microenvironments and immune niches provide additional forms of amino acid constraint. Quantitative measurements show that nutrient availability in tumors is heterogeneous, amino acid acquisition is a major determinant of cancer-cell adaptation, and amino acid catabolism in immune niches can engage GCN2-dependent T-cell programs [113,114,115,116]. Alternative translation and deprivation-induced substitutions can create altered protein products or shared antigens with therapeutic implications [77,78,117,118]. Whether BCAA-specific decoding phenotypes occur in tumors will depend on local supply, transporter expression, aaRS activity, tRNA pools, and initiation load.

A translational mechanism becomes physiologically persuasive only when the causal chain is measured in the same tissue and time window: dietary or disease-associated flux change; altered aminoacyl-tRNA state; codon-specific pausing or collision; signaling or substitution; and a protein- or organism-level phenotype. If the middle measurements are omitted, many other explanations remain open: endocrine signaling, energy balance, altered BCAA catabolism, and secondary changes in other amino acids.

## 9. Experimental Roadmap

The next stage should prioritize matched, time-resolved measurements rather than another isolated endpoint. Graded restriction—not only complete removal—of Leu, Val, and Ile should be compared in the same cell type. Early points should capture charging and ribosome states before transcriptional adaptation; later points should assess proteostasis and recovery. Medium and serum amino acids, cell density, harvesting method, and recovery intervals require explicit reporting. Given sex-, age-, and tissue-specific differences in BCAA responses and translational fidelity, these variables should be reported and, where feasible, tested directly [84,85,107,110]. Because aminoacyl-tRNA ester bonds are labile, the interval from sampling to acid fixation and all pH and temperature conditions must be recorded. Periodate-based RT-qPCR should report which anticodon isoacceptors and isodecoders are pooled by each primer set, distinguish relative aminoacylated-tRNA abundance from an absolute charged fraction, and measure total tRNA abundance in parallel [12].

Metabolomics should quantify extracellular uptake, intracellular pools, and isotope-resolved flux [19,22], paired with isoacceptor-resolved tRNA measurements. Conventional periodate-based assays and acid–urea separation assess aminoacylation but do not, without additional validation, establish the identity of the attached amino acid. Correct charging and misacylation therefore require amino acid-specific measurements. Modification-induced misincorporation tRNA sequencing (mim-tRNAseq) can quantify isoacceptor abundance and modification status [119]. Nanopore sequencing of intact aminoacylated tRNAs can combine sequence information with charging-state analysis and has shown pairwise amino acid discrimination using synthetic substrates [120]. However, isoleucyl-tRNAs were poorly distinguished from other hydrophobic aminoacyl-tRNAs, and absolute charging estimates were affected by sampling bias. Direct identification of Val-tRNA^Ile^ in mammalian samples therefore requires validated substrate discrimination and orthogonal confirmation. Cell-type-resolved tissue analysis must also establish that dissociation, fractionation, or spatial processing preserves the in situ charging state by controlling the interval to quenching and the pH and temperature throughout processing.

Ribosome profiling should include calibrated monosome and disome footprints. Reports should specify harvesting and flash-freezing, cycloheximide use, footprint-length selection, A- and P-site assignment, library normalization, and replicate handling. Measurements should meet the operational criteria in Table 1 and distinguish collision formation from its resolution and clearance. Position-controlled reporters can separate codon identity from local sequence and 5′ position, while mRNA abundance and half-life control for codon-dependent stability [15,40,92,93]. Calibrated run-off or single-mRNA imaging can estimate initiation and elongation separately and should be considered when polysome profiles are ambiguous [40,41].

Signaling analyses should resolve mTORC1, GCN2–eIF2α–ATF4, ZAKα–p38/JNK, and transcript-local EDF1–GIGYF2–4EHP responses in matched samples. Perturbations of the ribosomal P-stalk [48,49,54] and GCN1 recruitment or GCN1–ribosome and GCN1–GCN2 interfaces [50,51,52,53,55,56,57] should be interpreted in light of stress-, species-, and system-specific evidence. Interface dependence alone does not identify a collision-specific input; collision-low controls and validated separation-of-function perturbations are needed where feasible (Section 7.2). ZAKα perturbation tests collision-responsive p38/JNK signaling [7,58,59,60], whereas ZNF598 perturbation tests ribosome recognition and 40S ubiquitination, with the caveat that canonical ribosome collision is not always required for ZNF598-dependent activity [8,61,62,64]. RNF25 involvement should be tested by genetic perturbation coupled to measurements of GCN2–eIF2α–ATF4 signaling; eS31 ubiquitination can provide an additional mechanistic endpoint. These are proposed tests of a distinct GCN1-linked ISR regulator: the starvation-related mechanism remains emerging, whereas the established eS31 mechanism concerns RNA damage and has not yet been demonstrated during BCAA deprivation [89,90]. ASCC factors test ribosome splitting and disassembly [63,64], PELO–HBS1L–ABCE1 test stalled-ribosome rescue [67,68], and NEMF–LTN1 test post-splitting nascent-chain processing [9,10,16,17,18,65,66]. These factors represent interacting but separable signaling, recognition, rescue, and quality-control branches rather than a single linear pathway. Each perturbation must therefore be evaluated for secondary effects on initiation, ribosome load, and collision frequency.

Beyond these signaling and surveillance perturbations, proteomics must quantify both protein amount and verified sequence changes, followed by endogenous activity, localization, and stability assays. Substitution searches should address Ile/Leu isobaricity, false discovery, genomic variants, standards, isotope tracing, and reporter and endogenous evidence [75,76,79]. Response-calibrated molar peptide fractions should be interpreted in light of differential completion and turnover before comparison with the incorporation-conditioned substitution fraction in Equation (2). Comparison with the event-level probability in Equation (1) additionally requires accounting for abortive exits. Pulsed proteomics and matched reporters should partition lower protein output among reduced initiation, slower elongation, mRNA decay, aborted synthesis, nascent-chain degradation, and mature-protein turnover. For Ile deprivation, IARS1 and Val provide mechanistic perturbations, but reporter rescue is not equivalent to proteome-wide functional rescue [11]. Table 4 summarizes the corresponding measurement chain and the overinterpretations it is designed to prevent.

## 10. Conclusions

In the mammalian systems reviewed here, amino acid limitation is not adequately represented by initiation control alone. mTORC1 inhibition and GCN2–eIF2α signaling lower ribosome demand, but translating ribosomes still encounter aminoacyl-tRNA pools that differ among isoacceptors and codons. In the systems examined here, Leu deprivation is often initiation-dominant, yet it can produce UUA-biased pausing and frameshifting in selected cancer cells. Val deprivation can create broad elongation bottlenecks. Across separate studies, Ile deprivation has been associated with selective slowing in mouse NIH3T3 cells and an IARS1-linked Ile-to-Val signal in exogenous reporter peptides in human fibroblasts. Val-tRNA^Ile^ formation by purified IARS1 in vitro supports the proposed misacylation mechanism, whereas the mechanism underlying the observed Ile-to-Met substitution signal remains unresolved.

The mechanistic links remain partly disconnected. Under BCAA deprivation, changes in monosome occupancy have not yet been linked directly to disome formation, collision recognition, splitting, rescue, and nascent-chain fate within a single experiment. Likewise, reporter or growth rescue observed under conditions associated with substitution does not establish either causal mediation by substitution or endogenous proteome-wide functional rescue. The decisive experiments will measure supply, charging, initiation load, ribosome state, sequence change, degradation, and function in the same system and time course.

The two-layer kinetic framework organizes these tests without treating collision as an alternative outcome of A-site decoding. The first layer distinguishes event-level probabilities of correct accommodation, substitution, or abortive exit from substitution fractions conditional on incorporation; the second describes conditional collision risk using local influx, dwell time, and initial spacing. Keeping protein quantity, sequence fidelity, retained function, and proteostasis cost separate should clarify when amino acid restriction is adaptive, tolerated, or harmful.

## Figures and Tables

**Table 1:** Operational definitions and evidence boundaries used in this review.

Term	Working Definition	Minimum Evidence	Not Enough Alone
Pausing	Longer relative dwell or occupancy at a defined site.	Normalized monosome profiling or a position-controlled reporter.	Raw read pileup, lower bulk synthesis, or one endpoint.
Stalling	Persistent delay that impedes elongation flux.	Run-off or transit kinetics, time-resolved reporters, or single-mRNA imaging.	Single-time-point occupancy or lower downstream density.
Queueing	Multiple ribosomes accumulate upstream of a slow leader.	Position-resolved disome/trisome footprints, toeprinting, or structural evidence.	A single pause or reduced downstream density.
Collision	A trailing ribosome contacts a leading ribosome in diagnostic geometry.	Validated disome/trisome footprints or structural geometry; formation separated from clearance.	Pausing, stalling, or uncalibrated disome abundance.
Quality-control engagement	Direct rescue, ubiquitination, splitting, RNA surveillance, or nascent-chain triage.	A pathway factor plus a direct product: ubiquitination, rescue, decay, or chain fate.	ATF4 or p38/JNK activation, lower protein, or collision alone.
Functional deficiency	Cognate charging cannot meet current ribosome demand despite detectable free amino acid.	Supply or flux, tRNA abundance and aminoacylation, amino acid identity where relevant, and ribosome demand or dwell time.	Medium depletion, amino acid concentration, ATF4, or mTORC1 alone.
Adaptive translation	A defined translation change improves function or fitness under stress.	Mechanism-specific perturbation or rescue, a functional outcome, and stress controls.	Reporter output, short-term growth, or an altered product alone.

**Table 2:** Translational phenotypes reported during BCAA deprivation.

Condition	Direct Evidence	Working Interpretation	Main Limitation
Leu deprivation	Mouse NIH3T3, 6 h: approximately 60% lower aminoacylated tRNA^Leu^(CAR/WAG) signal relative to full-medium controls, with tRNA^Leu^(UAA) largely spared; only CUU showed a significant dwell-time increase [12]. Human HEK293T, HeLa, and HCT116 cells showed little Leu-codon pausing [5]. Selected human cancer cells, 48 h: limited tRNA^Leu^(UAA) availability, UUA-biased pausing, and preferential +1 frameshifting [43].	Usually initiation-dominant in acute systems; isoacceptor- and codon-specific effects vary by cell context and duration.	CAR/WAG measurements pool distinct anticodon isoacceptors and unresolved isodecoders; the ~60% decrease is a relative signal change. Acute NIH3T3 and prolonged cancer-cell studies are not directly comparable.
Val deprivation	Mouse NIH3T3, 6 h: broad Val-tRNA charging loss and slower decoding at all four Val codons [12].	A distributed elongation limit shaped by translation demand and recycling.	The 5′ bottleneck remains untested.
Ile deprivation	Mouse NIH3T3, 6 h: genomic tRNA^Ile^(AAU)/mature IAU group charging ↓; AUU/AUC slowing > AUA [12]. Human fibroblasts and purified IARS1: in vitro Val-tRNA^Ile^ formation and cellular Ile-to-Val reporter peptides [11].	Isoacceptor-selective slowing plus distinct sequence-change routes.	In vitro and cellular evidence are distinct; two patient lines shared one genotype; mass spectrometry covered two reporter peptides; endogenous rates remain unknown.
Combined Leu/Ile deprivation	Mouse NIH3T3, 6 h: strongest inhibition of selected mTORC1 readouts; weakest ISR activation; mild codon effects; only AUU increased significantly [12].	Lower ribosome demand may mask dual elongation defects.	Val was present; acute NIH3T3 study.
Triple BCAA deprivation	Mouse NIH3T3, 6 h: greatest O-propargyl-puromycin decrease; Val-dominant pausing and a 5′ density shift [12].	The most limiting route may dominate rather than add.	Acute deprivation in one mouse cell line.

Note: None of the cited BCAA studies directly measured disomes, ribosome collisions, or RQC engagement. ↓ denotes a decrease.

**Table 3:** Protein-sequence changes during BCAA limitation: established routes and unresolved mechanisms.

Route	Error Stage	BCAA-Linked Evidence	What Remains to Test
Misacylation	An aaRS loads the wrong amino acid onto a cognate tRNA.	Purified IARS1 generated Val-tRNA^Ile^ in vitro; Ile-restricted cells yielded *IARS1*-genotype-dependent Ile-to-Val peptides from an exogenous reporter [11].	Demonstrate Val-tRNA^Ile^ in deprived cells; validate reporter and endogenous peptides with standards; map target function.
Unresolved Ile-to-Met substitution	This substitution could originate from tRNA misacylation or near-cognate codon recognition at the ribosome; the responsible route remains unresolved.	Ile-to-Met reporter peptides occurred both in healthy cells and in IARS1-deficient cells that retained residual IARS1 activity; the responsible aaRS/tRNA route is unresolved [11].	Identify the amino acid on the relevant tRNA; combine aaRS/tRNA perturbations, codon reporters, and validated peptides.
Frameshifting	The reading frame shifts during elongation.	Selected cancer cells: Leu limitation, tRNA^Leu^(UAA) availability ↓, UUA pausing, and +1 frameshifting [43].	Use frame-specific reporters, codon/tRNA rescue, mRNA controls, and direct product validation.

Note: ↓ denotes a decrease.

**Table 4:** Measurements required to test the proposed causal chain.

Layer	Preferred Measurements	Question Answered	Overinterpretation Avoided
Supply and flux	Metabolomics; isotope tracing; uptake, proteolysis, and catabolism.	How do supply, use, and recycling change?	Concentration alone proves functional limitation.
tRNA state	Rapid quenching; isoacceptor abundance and aminoacylation; validated amino-acid-specific analysis with orthogonal confirmation; aaRS activity and editing.	Which tRNAs change in aminoacylated fraction, and which amino acids are attached?	A charging signal proves correct amino acid identity; all synonymous codons behave alike.
Ribosome state	Calibrated monosome and disome profiling; controlled harvesting; A- and P-site assignment; position-controlled reporters.	Is dwell time longer? Do queues or collisions form, and how quickly are they cleared?	Monosome occupancy proves collision or RQC.
Signaling and quality control	GCN2–eIF2α–ATF4; mTORC1; EDF1–GIGYF2–4EHP; ZAKα; ubiquitination; splitting or rescue; RQC perturbation.	Which global, local, rescue, or nascent-chain branches engage?	Interface dependence proves a collision-specific input; a translation phenotype proves pathway activation.
Protein outcome	Pulsed proteomics; response-calibrated substitution peptides and standards; activity or localization; aggregation or degradation.	Are amount, fidelity, and function coupled?	Higher reporter output proves functional rescue.
Physiology	Tissue-matched time course; cell-state phenotyping; dietary recovery; organismal endpoints.	Is the molecular route causal in vivo?	A dietary phenotype proves a cell-culture mechanism.

## Data Availability

No new datasets were generated. Publicly available source data from Worpenberg et al. [12] were inspected to verify quantitative statements in Section 3.1, Section 3.2 and Section 3.3, Table 2, and Figure 2. The Leu relative-signal estimate was checked against the Charged entries for untreated Leu deprivation in Figure_4_tRNA_charging/Data/All_Replicates.xlsx and the associated plotting script; it is not an estimate of a percentage-point change in absolute charging. The underlying data and analysis resources are available through the Gene Expression Omnibus (GSE291652 and GSE291653), ProteomeXchange (PXD067949), GitHub (https://github.com/naef-lab/Worpenberg_2025_GB_BCAA_starvation/ (accessed on 8 September 2026)), and Zenodo (https://doi.org/10.5281/zenodo.17035796).

## Abbreviations

The following abbreviations are used in this manuscript:

4EHP	eIF4E homologous protein
aaRS	aminoacyl-tRNA synthetase
ABCE1	ATP-binding cassette subfamily E member 1
ADAT2/3	adenosine deaminase acting on tRNA, subunits 2 and 3
ASCC	activating signal cointegrator 1 complex
ATF4	activating transcription factor 4
BCAA	branched-chain amino acid
BCAT	branched-chain aminotransferase
BCAT2	branched-chain aminotransferase 2
BCKD	branched-chain α-keto acid dehydrogenase
CTNNA1	catenin alpha 1
EDF1	endothelial differentiation-related factor 1
eIF2α	eukaryotic initiation factor 2α
eS31	ribosomal protein eS31
GATOR	GTPase-activating protein activity toward Rags
GCN1	general control nonderepressible 1
GCN2	general control nonderepressible 2
GFP	green fluorescent protein
GIGYF2	GRB10-interacting GYF protein 2
HBS1L	HBS1-like translational GTPase
I34	inosine at transfer RNA position 34
IARS1	cytoplasmic isoleucyl-tRNA synthetase 1
ISR	integrated stress response
IUPAC	International Union of Pure and Applied Chemistry
JNK	c-Jun N-terminal kinase
*KRAS*	KRAS proto-oncogene, GTPase
LARS1	leucyl-tRNA synthetase 1
LAT1	L-type amino acid transporter 1
LTN1	listerin E3 ubiquitin protein ligase 1
MAP3K20	mitogen-activated protein kinase kinase kinase 20 (ZAK)
mim-tRNAseq	modification-induced misincorporation tRNA sequencing
mRNA	messenger RNA
mTORC1	mechanistic target of rapamycin complex 1
NEMF	nuclear export mediator factor
PELO	pelota mRNA surveillance and ribosome rescue factor
QC	quality control
RACK1	receptor for activated C kinase 1
RagD	Ras-related GTP-binding protein D
Ribo-seq	ribosome profiling
RNA-seq	RNA sequencing
RNF25	ring finger protein 25
RQC	ribosome-associated quality control
RSR	ribotoxic stress response
RT-qPCR	reverse-transcription quantitative polymerase chain reaction
SAMP8	senescence-accelerated mouse prone 8
SLFN11	Schlafen family member 11
TFEB	transcription factor EB
tRNA	transfer RNA
ZAKα	α isoform of mitogen-activated protein kinase kinase kinase 20
ZNF598	zinc finger protein 598
Ψ	pseudouridine
